# Resting-state functional connectivity changes following audio-tactile speech training

**DOI:** 10.3389/fnins.2025.1482828

**Published:** 2025-04-29

**Authors:** Katarzyna Cieśla, Tomasz Wolak, Amir Amedi

**Affiliations:** ^1^The Baruch Ivcher Institute for Brain, Cognition, and Technology, The Baruch Ivcher School of Psychology, Reichman University, Herzliya, Israel; ^2^The Ruth and Meir Rosenthal Brain Imaging Center, Reichman University, Herzliya, Israel; ^3^World Hearing Centre, Institute of Physiology and Pathology of Hearing, Warsaw, Poland

**Keywords:** speech comprehension, tactile aid, multisensory training, fMRI, resting-state functional MRI, cochlear implants

## Abstract

Understanding speech in background noise is a challenging task, especially when the signal is also distorted. In a series of previous studies, we have shown that comprehension can improve if, simultaneously with auditory speech, the person receives speech-extracted low-frequency signals on their fingertips. The effect increases after short audio-tactile speech training. In this study, we used resting-state functional magnetic resonance imaging (rsfMRI) to measure spontaneous low-frequency oscillations in the brain while at rest to assess training-induced changes in functional connectivity. We observed enhanced functional connectivity (FC) within a right-hemisphere cluster corresponding to the middle temporal motion area (MT), the extrastriate body area (EBA), and the lateral occipital cortex (LOC), which, before the training, was found to be more connected to the bilateral dorsal anterior insula. Furthermore, early visual areas demonstrated a switch from increased connectivity with the auditory cortex before training to increased connectivity with a sensory/multisensory association parietal hub, contralateral to the palm receiving vibrotactile inputs, after training. In addition, the right sensorimotor cortex, including finger representations, was more connected internally after the training. The results altogether can be interpreted within two main complementary frameworks. The first, speech-specific, factor relates to the pre-existing brain connectivity for audio–visual speech processing, including early visual, motion, and body regions involved in lip-reading and gesture analysis under difficult acoustic conditions, upon which the new audio-tactile speech network might be built. The other framework refers to spatial/body awareness and audio-tactile integration, both of which are necessary for performing the task, including in the revealed parietal and insular regions. It is possible that an extended training period is necessary to directly strengthen functional connections between the auditory and the sensorimotor brain regions for the utterly novel multisensory task. The results contribute to a better understanding of the largely unknown neuronal mechanisms underlying tactile speech benefits for speech comprehension and may be relevant for rehabilitation in the hearing-impaired population.

## Introduction

Resting-state functional magnetic resonance imaging (rsfMRI) is based on the premise that spontaneous low-frequency oscillations in the brain, while, at rest, it can reveal its functional organization and alterations, without the need for the participant to engage in a specific task (Biswal et al., 1995; Raichle, 2009). At the same time, the signals obtained from task-MRI and rsfMRI are likely to stem from similar structural connections and neuronal processes (Ngo et al., 2022). In addition, in resting-state protocols, the oscillations of the blood-oxygenation-level–dependent (BOLD) signal offer a signal-to-noise ratio up to three times higher than the signal increases observed during task-related activities (Fox and Greicius, 2010). Using a measure of functional connectivity (FC), which represents signal correlations among remote brain regions, rsMRI has been shown to reveal language networks with high sensitivity (e.g., Lemée et al., 2019; Tie et al., 2014; Zhu et al., 2014; Branco et al., 2016; Sun et al., 2019; Liu et al., 2021). Considerable FC changes have been reported as a result of several-minute- or several-week-long cognitive or motor interventions, as well as aging (e.g., Katsuno et al., 2022; Maruyama et al., 2018; Cao et al., 2014; Farrens et al., 2023; Kawata et al., 2022; Bamidis et al., 2014). It has been proposed that, to gain a deeper understanding of changes in brain organization, it is essential to consider functional brain networks.

In the current study, we used the rsfMRI method specifically to investigate changes in functional connectivity following speech comprehension training, combining congruent auditory and vibrotactile inputs on the fingertips.

Audio-vibrotactile interactions are relatively common in our everyday lives, for example, when receiving tactile feedback from a ringing phone, when steering wheel, or while playing computer games. The combination and integration of these two sensory signals seem natural and inherent (Van Békésy, 1959), which is possibly due to the significant similarities between the two sensory modalities, including their ability to encode the very same oscillatory patterns within an overlapping frequency range using mechanoreceptors. Specifically, for the tactile sense, the Pacinian corpuscles in the skin respond to vibrations of up to 700–1,000 Hz, while the auditory system encodes vibrations in the air through inner hair cells between 20 Hz and 20 kHz (Bolanowski et al., 1988; Schnupp et al., 2011).

In several published studies, congruent vibrotactile inputs have been shown to successfully enhance music perception (e.g., Russo et al., 2012; Sharp et al., 2019), as well as the localization of sounds in space (Gori et al., 2014; Occelli et al., 2009; Snir et al., 2024). Another specific use of low-frequency vibrations has been to improve speech comprehension. This approach was originally developed to assist the deaf population, with tactile aids designed to convey different features of speech signals via the skin (e.g., Galvin et al., 1991; Weisenberger and Percy, 1995). In a series of recent studies by our lab and others, it has been shown that improvement of speech perception is also possible in both typically hearing and hearing-impaired individuals using cochlear implants, with continuous speech-extracted vibrations delivered on the fingertips (Cieśla et al., 2019, 2022; Fletcher et al., 2018; Fletcher et al., 2019; Huang et al., 2017; Schulte et al., 2023; Rǎutu et al., 2023). Most authors implemented some form of manipulation to the speech signal to mimic the acoustic conditions that are most challenging for the hearing-impaired population, such as speech distortions and/or simultaneous background noise.

This rsfMRI study complements our parallel task-fMRI study (manuscript under review; preprint at Cieśla et al., 2024), with participants performing speech comprehension tasks inside an MRI scanner before and after dedicated audio-tactile speech comprehension training. The speech signals consisted of distorted sentences (vocoded to resemble speech delivered through a cochlear implant system), presented alongside speech background noise. The tactile vibrations corresponded to low frequencies (< 200Hz) extracted directly from the speech inputs using an adjusted STRAIGHT algorithm (Kawahara et al., 1999). We demonstrated enhanced comprehension following audio-tactile training, which aligns with findings from several other studies that utilized audio–visual speech stimuli, as well as those showing improved intelligibility of distorted speech with practice time (e.g., Casserly and Pisoni, 2015; Erb et al., 2013; Guediche et al., 2014; Bernstein et al., 2013). The results suggest that the speech-extracted tactile signal can potentially work in a manner similar to the visual signal (such as lip-reading) through temporal synchronization with auditory inputs (O'Sullivan et al., 2021; Schulte et al., 2023).

Here, we speculated that training-induced neuronal changes would also manifest in resting-state functional connectivity (FC). Specifically, brain networks were identified using independent component analysis (ICA) and tested for functional connectivity before and after audio-tactile training. Based on our previous behavioral findings indicating successful multisensory integration of speech stimuli (Cieśla et al., 2019, 2022), we expected to observe enhanced connectivity between the auditory and the somatosensory resting-state networks, as well as within the language network (Price, 2012; Hertrich et al., 2020). This finding includes multisensory areas such as the posterior superior temporal sulcus (pSTS), the angular gyrus/supramarginal gyrus (AG/SMG), and the lateral occipital cortex (LOC), which have been shown in the literature for audio-visual and audio-tactile integration (e.g., Renier et al., 2009; Kassuba et al., 2013; Landelle et al., 2023; King et al., 2019; Nath and Beauchamp, 2012; Hickok et al., 2018). The applied outcome measure was the difference in functional connectivity patterns between two resting-state fMRI sessions, before and after audio-tactile speech comprehension training.

## Materials and methods

The study took place at the ELSC Neuroimaging Unit in Jerusalem, Israel. All participants gave informed consent and received compensation for their participation. The study procedures complied with the World Medical Association (2013) and received approval from the Ethics Committee of the Hadassah Medical Center in Jerusalem (protocol no. 353-18.1.08).

### Participants

The reported data were acquired from 20 adult healthy individuals (8 male/12 female, 26.8+/−4.2), all of whom were right-handed and had no history of neurological or neurodevelopmental impairments.

### Experimental procedures

#### Behavioral experimental procedures

Between the two resting-state fMRI sessions, the participants were tested twice (before and after the training) on their vocoded speech-in-noise comprehension. There were three test conditions: a) auditory sentences (A), b) auditory sentences with congruent tactile vibrations delivered to two fingertips of the right hand (audio-tactile congruent, ATc), and c) auditory sentences accompanied with non-congruent tactile vibrations on the fingertips (audio-tactile non-congruent, ATnc). For each test condition, an individual speech reception threshold (SRT)—i.e., the SNR between the target sentence and the background noise required for 50% understanding—was determined. The used sentences were derived from the Hearing-In-Noise-Test (HINT) database (Nilsson et al., 1994) and vocoded to resemble stimulation through a cochlear implant (Walkowiak et al., 2010). In the multisensory test conditions, low-frequency vibrations (< f0 of the spoken sentence, i.e., 200 Hz) were extracted from either the concurrently presented auditory sentence (audio-tactile congruent, ATc) or from another randomly selected sentence from the HINT database (audio-tactile non-congruent, ATnc) and delivered to the index and middle fingers of the right hand through an in-house developed device (Cieśla et al., 2019). Between the two SRT testing sessions, the participants took part in a 30–45 min training session. They were asked to repeat 148 HINT sentences, one by one, with (group 1) or without (group 2) accompanying congruent tactile vibrations delivered to the fingertips, at individually determined SRTs. The training session continued until all 148 sentences were repeated correctly without feedback. The results of the behavioral study, including pre- and post-training speech comprehension measurements, are published elsewhere (Cieśla et al., 2022). The resting-state fMRI study was conducted in a subgroup of participants who underwent audio-tactile speech comprehension training.

#### Resting-state fMRI data acquisition

The resting-state fMRI data were acquired twice using a 3T Skyra MRI scanner by Siemens and a 32-channel head coil, before and after the training, during a 10:11 min session. The parameters were as follows: TR =1.5 s, TE = 32.6 ms, voxel size = 2 × 2 × 2 mm, 72 slices, flip angle = 78, FOV = 192 mm, and bandwidth = 1,736 Hz. The participants had their eyes open and were asked to fixate on a cross presented on the screen. In addition, a high-resolution T1 MR sequence was collected with the following parameters: TR = 2.3 s, TE = 2.98 ms, IT = 900 ms, matrix size = 256 × 256, voxel size = 1 × 1 × 1 mm, 160 slices, and bandwidth = 240Hz/voxel.

### Data analysis

#### Resting-state fMRI data analysis

To evaluate functional connectivity patterns between brain regions, the CONN 22a (Connectivity Toolbox, https://web.conn-toolbox.org/) software was used for data analysis. Preprocessing of the functional data included the following: slice timing correction, motion correction, scrubbing, linear detrending, band-pass filtering (0.008 Hz < f < 0.09 Hz), co-registration to individual T1 structural scans, surface-based spatial normalization (https://surfer.nmr.mgh.harvard.edu/), and spatial smoothing (6 mm Gaussian kernel).

Denoising was applied using default settings, including the regression of potential confounding effects characterized by white matter time series (five CompCor noise components), cerebrospinal fluid (CSF) time series (five CompCor noise components), motion parameters and their first-order derivatives (12 factors), outlier scans (below 25 factors), and linear trends (two factors) within each functional run. This was followed by bandpass frequency filtering of the BOLD time series between 0.008 Hz and 0.09 Hz. The CompCor noise components within the white matter and CSF were estimated by computing the average BOLD signal and the largest principal components orthogonal to the BOLD average, motion parameters, and outlier scans within each participant's eroded segmentation masks. Based on the number of noise terms included in this denoising strategy, the effective degrees of freedom of the BOLD signal after denoising were estimated to range from 59.8 to 125 (average 94.7) across all participants.

**Figure 1:**
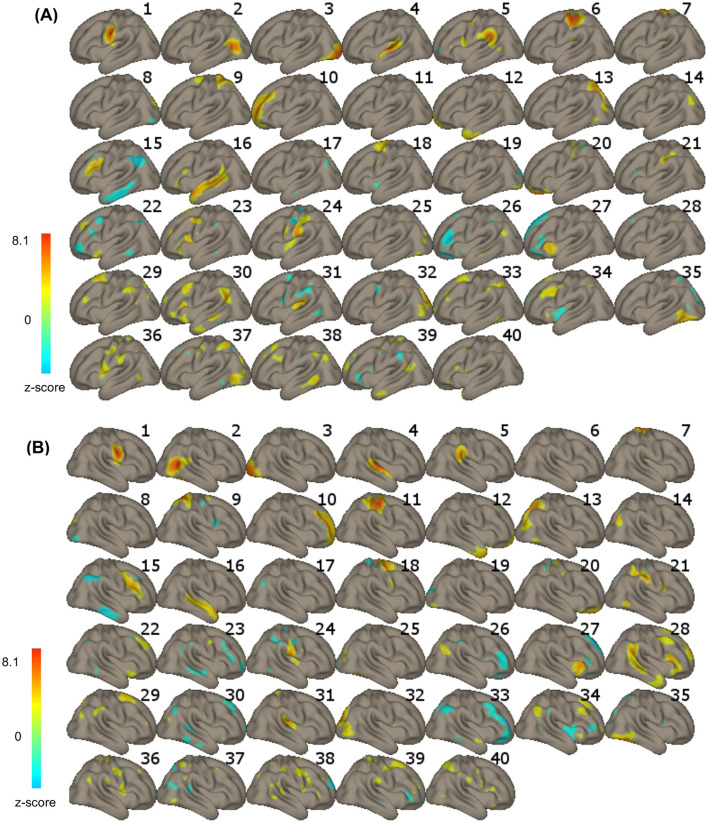
**(A)** Left-hemisphere and **(B)** right-hemisphere aspects of the 40 independent components (ICs) revealed in the IC analysis. The color bar represents voxel-to-voxel correlation z-values (threshold at z = 2); yellow to red in the color bar indicates a positive correlation, and green to blue indicates a negative correlation.

**Figure 2:**
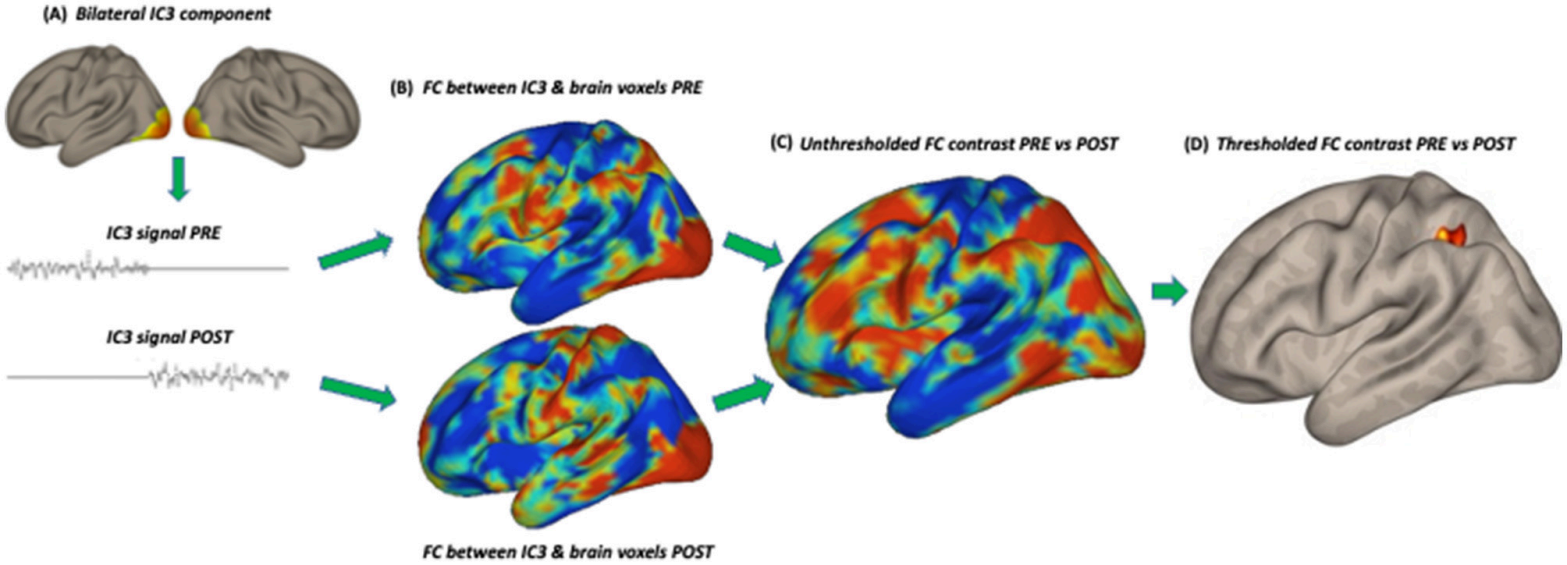
Subsequent steps of the statistical analysis: **(A)** extraction of the signal from an independent component in the session before (PRE) and after (POST) training, **(B)** functional connectivity (FC) analysis in the PRE and POST sessions separately with the signal in all brain voxels, **(C)** unthresholded FC comparison between PRE and POST, **(D)** thresholded FC comparison between PRE and POST. IC3 (early visual cortex) is used as an example.

**Figure 3:**
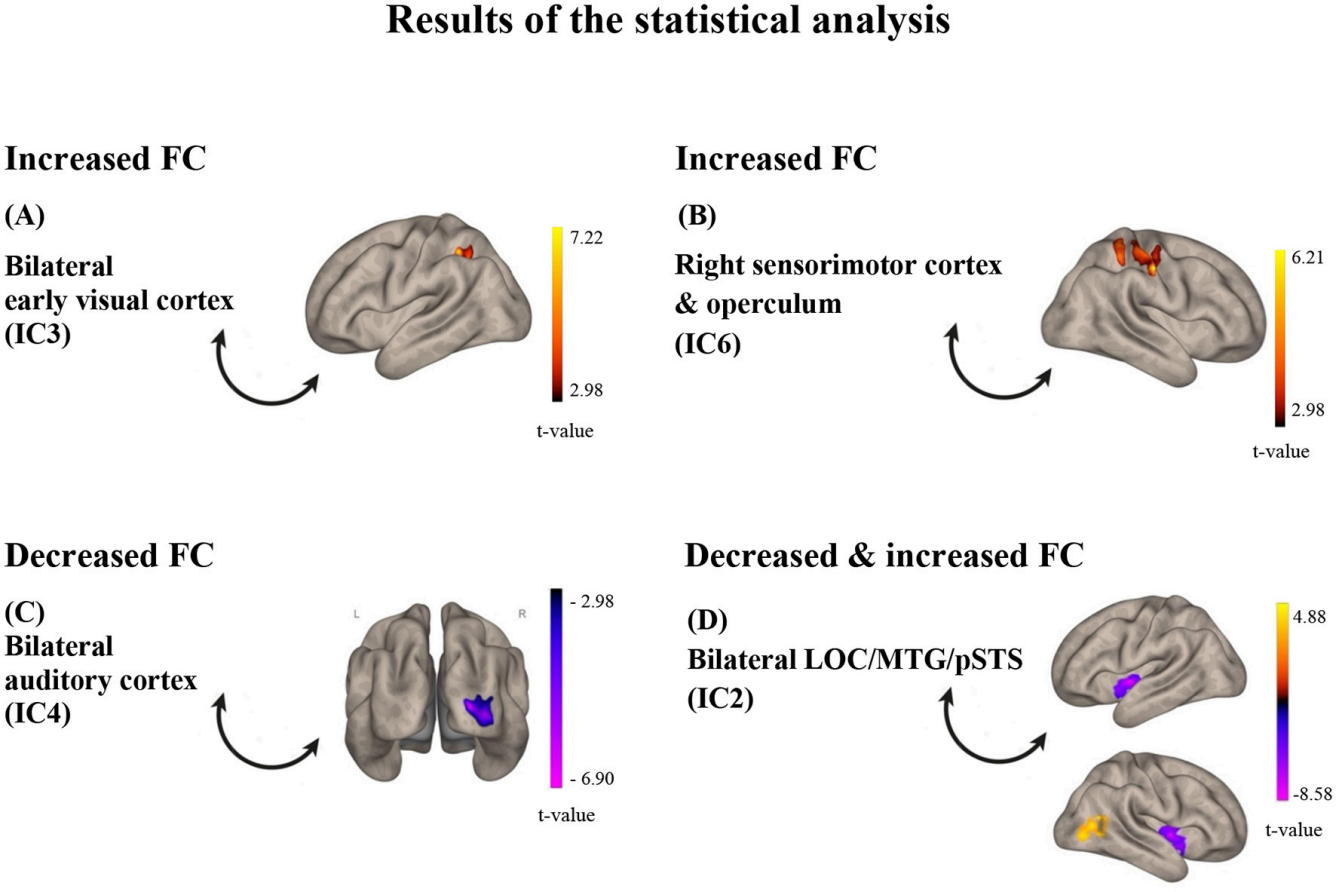
Results of the statistical analysis. **(A)** for IC3, FC was found increased with a cluster in the left SPL/aSMG/PostCG after training; **(B)** for IC6, FC was found decreased with the right sensorimotor cortex after training; **(C)** for IC4, FC was found decreased with the right primary visual cortex; **(D)** for IC2, FC was found both increased with a cluster in the right LOC/pMTG (red-yellow) and decreased with the insula (purple-pink) after training. The color bars represent *t*-values of the statistical comparison of functional connectivity POST vs. PRE training; *t*-values above zero (red-yellow) indicate increased FC after training, while *t*-values below zero (purple-pink) indicate decreased FC after training. IC, Independent Component; LOC, lateral occipital cortex; MTG, middle temporal gyrus; pSTS, posterior superior temporal sulcus.

Group ICA) was used for statistical analysis. ICA is a data-driven approach that investigates multiple voxel-to-voxel interactions of several networks in the brain simultaneously. Based on the acquired signal, ICA separates noisy components from those representing neuronal activation in the form of resting-state networks. Networks represent sets of regions with voxel-by-voxel correlated BOLD signal fluctuations (Smitha et al., 2017; Rosazza et al., 2012). Using the CONN software, we applied a G1 FastICA + GICA3 back-projection procedure with dimensionality reduction set at 64 (64 principal components) and the number of independent components set at 40 (components optimized for orthogonality; 40 is the default value), based on the recorded BOLD signal combined across all participants. Components comprising the language (including auditory cortex), visual, and sensorimotor networks were selected for further analysis (Figure 1, ICAs 1–6, 11, 16). For each of the separately selected eight components, functional connectivity (FC) was estimated with all other voxels in the brain. Next, FC was compared between the PRE and POST training sessions for each component using *t*-tests. See the subsequent steps of the analysis in Figure 2. Specific outcome brain regions were identified that showed increased FC after the training compared to before the training (Figure 3). The statistical analysis used permutation with the following parameters: voxel-threshold with a *p*-value of < 0.01 and cluster-mass p-FDR corrected *p*-value of < 0.05. The regions were labeled using the Harvard-Oxford Atlas.

## Results

### Resting-state fMRI functional connectivity changes after training in the auditory, sensorimotor, and visual systems

Of the eight comparisons of FC before and after training, four turned out to be statistically significant (Figure 3). Specifically, resting-state functional connectivity increased after, compared to before, the audio-tactile speech comprehension training between bilateral clusters encompassing parts of the lateral occipital cortex (LOC), angular gyrus (AG), posterior medial temporal gyrus (pMTG), and posterior superior temporal sulcus (pSTS; IC2; including functional areas, such as MT +/−44 −68 2 and extrastriate body area (EBA) –/+ 50 −70 5; https://neurosynth.org/), as well as LOC/pMTG/AG on the right side, indicating increased internal connectivity within this network. In addition, increased connectivity was observed between bilateral clusters encompassing parts of the occipital pole (OP), LOC, lingual gyrus (LG), and intracalcarine cortex (ICC; early visual cortex; IC3), as well as a hub representing the left superior parietal lobe (SPL), anterior supramarginal gyrus (aSMG), and postcentral gyrus (PostCG). Increased connectivity was observed in a right-hemisphere cluster encompassing parts of PostCG, precentral gurus (PreCG), and SPL (sensorimotor cortex and operculum, IC6), with an almost overlapping area. At the same time, decreased functional connectivity after training was observed between bilateral clusters encompassing parts of iLOC, AG, pMTG, and pSTS (IC 2) and bilateral anterior insula, as well as between bilateral clusters encompassing parts of the superior temporal gyrus (STG), temporal pole (TP), polar pole (PP), SMG (auditory cortex, IC 4), and OP and LOC on the right side (early visual cortex). The results are depicted in Figures 1–3.

## Discussion

As reported in a series of our previous studies using an in-house setup, congruent low-frequency speech-extracted vibrations on the fingertips can improve auditory speech perception in difficult acoustic conditions by an average of 4–6 dB SNR (Cieśla et al., 2019, 2022). Interestingly, training did not increase this tactile benefit any further, suggesting that only to a certain degree one can use tactile information naturally. In addition, audio-tactile training was found to be more efficient than auditory training, leading to comparable improvement but in more difficult acoustic conditions (Cieśla et al., 2022). In this study, we present resting-state fMRI findings from a subgroup of participants trained using audio-tactile speech stimulation. The results provide insights into the brain networks likely engaged in the new experience of audio-tactile speech perception and insights that promote training-related improvements in comprehension.

The revealed functional connectivity (FC) results showed several effects when the pre- and post-training resting-state networks were compared, which can be summarized as follows. First, we observed a shift in the functional connectivity of the lateral occipital cortex (LOC) from increased FC with the bilateral dorsal anterior insula to increased internal connectivity (i.e., between the right LOC and itself). Second, there was a change in the functional connectivity of the early visual system, shifting from one with the auditory system before training to one with a hub in the left somatosensory/multisensory association cortex afterward. Third, the sensorimotor system in the right hemisphere showed increased internal FC after training. While the effects involving the auditory and sensorimotor systems were hypothesized following an audio-tactile intervention, the engagement of the early visual system was not. Interestingly, this system was also observed in a parallel task-fMRI study by our lab [under review; see also a preprint at: Cieśla et al., 2024]. In the previously mentioned study, the participants were asked to repeat distorted sentences inside the MRI scanner, before and after speech comprehension training, with and without tactile vibrations delivered to the fingertips. The effects observed for the trained audio-tactile speech condition partially correspond to those revealed in the current resting-state analysis, including significant involvement of the visual system in both learning and multisensory integration, especially in the early visual regions and LOC. This finding further suggests that the signals obtained from task-fMRI and rsfMRI are likely to stem from similar structural connections and neuronal processes (Ngo et al., 2022).

We speculate that the revealed “visual” regions (both early V1–V2 and LOC) might represent parts of a network typically reported for audio-visual speech comprehension (e.g., Hertrich et al., 2020; Beer et al., 2013; Nath and Beauchamp, 2012; Hauswald et al., 2018). The involvement of these regions indicates that the brain functional system engaged in the utterly novel task of processing audio-tactile speech might be first built upon an existing blueprint for connectivity. In the current study, the speech task required comprehension of sentences in a non-native language that were also vocoded (resembling cochlear implant stimulation) and presented against background speech noise, all rendering the task rather challenging. In everyday situations, when exposed to ambiguous speech, either distorted or occurring in a challenging acoustic environment, we naturally search for informative visual cues, such as from lip-reading. These audio-visual associations develop in the early years of life and are practiced on a regular basis throughout lifetime (Hickok et al., 2018; Dopierała et al., 2023). Furthermore, the involvement of the early visual cortex has been previously reported for auditory and tactile (e.g., Braille reading) language tasks as well. Several studies have demonstrated this effect in both blind and sighted individuals, indicating that early visual regions have the capacity for amodal language processing (Amedi et al., 2003; Bedny et al., 2015; Reich et al., 2011; Seydell-Greenwald et al., 2023; Merabet et al., 2008). In this study, after the audio-tactile speech training, once it became apparent that no informative visual cues were available during task performance, the pre-existing (and potentially automatically engaged in the context of speech perception) connectivity between the auditory system and the early visual system decreased (Panel C in Figure 2).

The other identified “visual” hub in the lateral occipital/temporal lobe encompassed regions such as the middle temporal visual area (MT), EBA, and LOC (Panel D in Figure 2). These brain areas, among other functions, can play a role in non-verbal communication, specifically in encoding lip movements, body gestures, facial expressions, and other language-related spatial cues (Kitada et al., 2014; Hickok et al., 2018; Van der Stoep and Alais, 2020). All these cues can facilitate comprehension in difficult acoustic conditions. Furthermore, the revealed adjacent pSTS is recognized as a key hub for multisensory integration, particularly for audio-tactile and audio-visual inputs. It specifically specializes in processing temporally changing signals, such as speech (King et al., 2019; Hertrich et al., 2020; Beer et al., 2013; Beauchamp et al., 2004). In a non-speech-specific context, the revealed area of the LOC has also been shown to have multisensory properties (although mainly audio-visual) and has been implicated in activities such as grasping and object recognition (e.g., Kassuba et al., 2013; Jao et al., 2015; Brand et al., 2020), as well as in response to passive vibrotactile stimulation on the hand—potentially related to the mechanism of object manipulation through increased connectivity with SI (Tal et al., 2016). During the speech comprehension training, the participants both listened to speech stimuli through headphones and had the fingertips of their right hand placed in the tactile device. This specific multisensory setup may also involve body awareness and reaching behavior, which could be reflected in resting-state functional network changes.

The functional connectivity of the LOC after training diminishes with the bilateral anterior insular cortex. This outcome may stem from the insula's role in various cognitive processes, particularly in the context of developing new skills or adapting to new environments (e.g., Gogolla, 2017; Zühlsdorff et al., 2023; Ardilla et al., 2014). Specifically, the revealed dorsal anterior part of the insula, through its multiple connections with the rest of the brain, has been found to participate in language processing and general learning (e.g., Ardilla et al., 2014; although see Woolnough et al., 2019), cognitive control, spatial attention tasks, and multimodal integration—as opposed to the social-emotional functions typically attributed to the posterior and ventral-anterior aspects of the insula (Kurth et al., 2010; Uddin et al., 2017). In addition, the anterior insula may play a role in bodily ownership, bodily self-awareness, and tactile perception (Wang et al., 2019). One suggested mechanism is through direct projections from the secondary somatosensory cortex to the insular cortex, which, in turn, innervates regions of the temporal lobe believed to be critical for tactile learning and memory (Kurth et al., 2010; Augustine, 2023). The decreased involvement of the insula possibly reflects progress in learning to use the newly available tactile inputs for improved speech comprehension.

Considering the role of the sensorimotor system, we demonstrate two additional effects. First, the applied audio-tactile speech training resulted in increased connectivity from the bilateral early visual cortex to a posterior parietal region on the side opposite to the vibrotactile stimulation. Part of the revealed hub can be considered the somatosensory association cortex, where the experienced low-frequency tactile signal may be analyzed for its temporal and speech features. Other tactile operations attributed to this area include Braille reading, tactile attention, shape recognition, and others (e.g., Kim et al., 2015; Bodegard et al., 2001; Li Hegner et al., 2010). At the same time, the superior parietal lobe—comprising 40% of the revealed cluster—receives inputs not only from the early sensory system, particularly the hand, but also from other modalities, which are further combined to inform behaviors, such as reaching or grasping (Sheth and Young, 2014; Goodale and Milner, 1992). Finally, the demonstrated left-hemisphere SMG (Panel A in Figure 2) plays a critical role in the phonological analysis of language inputs, while also having multisensory properties (e.g., Oberhuber et al., 2016).

The increased functional connectivity after training between the early visual and the sensorimotor system/multisensory is not a straightforward result. It might reflect a mechanism similar to the one reported in some other studies on tactile perception, although in the opposite direction (Tal et al., 2016; Merabet et al., 2007). Specifically, Tal et al. showed deactivation of early visual regions (e.g., V1–V2) when participants experienced passive, delicate brushing of various body parts—although a single-case EcoG study by Gaglianese et al. (2020) showed positive involvement of V1 during brushing. In contrast, Merebet et al. found active engagement of V1 in estimating roughness or inter-dot spacing in tactile patterns. This study indicates that the responsiveness of early visual regions during tactile stimulation might be task-specific and could reflect processes such as motion processing, spatial analysis, or tactile-to-visual remapping of inputs. As suggested by some authors, the increased FC with early visual areas after training and its general role in the current experiment might also be mediated by the visualization of the content of the speech signal or the vibrating devices (e.g., Reiner, 2008).

In addition, after training, we observed increased internal connectivity within the right-hemisphere sensorimotor system (ipsilateral to the tactile finger stimulation). The cluster encompasses the primary (SI), secondary (SII, operculum), and somatosensory association cortices, including areas representing the fingers, as well as the corresponding motor cortex. This finding was unexpected but may be related to several mechanisms. First, although responses to tactile stimuli are found to be mainly contralateral (and especially in SI), both animal and human neuroimaging studies have shown modulation of responses in ipsilateral SII (and to a lesser extent, also in SI), possibly through dense transcallosal connectivity. Moreover, the bilateral sensorimotor association cortex in the posterior parietal cortex is the area where inputs from both hands become integrated (Pala and Stanley, 2022; Tamè et al., 2012; Hlushchuk and Hari, 2006). The sensorimotor cortex is also densely connected with the bilateral motor cortex, specifically the regions representing hands, which enables the successful manipulation of objects. The sensorimotor interactions involve the motor cortex responding to tactile stimulation, as well as the sensory cortex receiving feedback from and regulating motor behavior (Mastria et al., 2023; Tamè et al., 2015; Bao et al., 2024). Further research is needed to understand why the observed effect was ipsilateral to the stimulated hand rather than contralateral.

In summary, the main results of the study highlight the role of the visual network, auditory system, sensorimotor system, and insula in the audio-tactile integration of speech information and learning using our specific multisensory setup. The findings are in partial agreement with our hypotheses— e.g., showing engagement of the early sensory systems, as well as the pSTS and SMG in multisensory integration—although no enhancement of direct connectivity was found between the auditory and the sensorimotor cortex. The lack of such an effect might reflect comparable tactile benefits for speech perception before and after training (Cieśla et al., 2022). The involvement of the visual system (LOC, early regions), potentially mediating auditory-to-tactile communication and thus the integration of inputs, indicates that functional network changes following perceptual learning might be initially constrained by the existing connections established through experience (Makin and Krakauer, 2023; Heimler and Amedi, 2020). Since the audio-tactile speech context is novel—never experienced in a lifetime or in evolution—unlike the well-trained audio-visual speech context, a longer training regime might be necessary to modify pre-existing connectivity patterns. This might, in turn, translate into greater benefits of adding tactile information. In addition, some of the observed results in the visual, sensorimotor, and insular networks can be interpreted within the framework of bodily awareness, grasping, and mental imagery, as the task required successful integration of inputs arriving from different spatial locations. One limitation of the current study is the lack of assessment of functional connectivity changes following auditory-only versus multisensory audio-tactile speech training, especially given our behavioral results showing its significant but less profound effect on learning (Cieśla et al., 2022).

## Data Availability

The raw data supporting the conclusions of this article will be made available by the authors, without undue reservation.
